# Treg dysregulation in the oxidative lung milieu: an emerging mechanistic link in interstitial lung disease associated with systemic autoimmune rheumatic diseases

**DOI:** 10.3389/fimmu.2026.1882853

**Published:** 2026-09-24

**Authors:** Yanan Li, Jinghua Wang, Hongsheng Sun, Min Fu

**Affiliations:** Department of Rheumatology, Shandong Provincial Hospital Affiliated to Shandong First Medical University (Shandong Provincial Hospital), Jinan, China

**Keywords:** immune imbalance, interstitial lung disease, oxidative stress, regulatory T cell, systemic autoimmune rheumatic disease

## Abstract

Systemic autoimmune rheumatic disease-associated interstitial lung disease (SARD-ILD) is a fatal complication, in which the progression of pulmonary fibrosis has been closely linked to immune microenvironment imbalance and oxidative stress. Regulatory T cells (Tregs), as a central component of immune tolerance, play a crucial role in suppressing excessive inflammation. Current evidence suggests that oxidative stress may contribute to Treg dysregulation through several interrelated mechanisms, including redox-sensitive regulation of Foxp3 expression and stability, disruption of IL-2 signaling, and remodeling of the inflammatory microenvironment. However, the majority of the mechanistic evidence is derived from experimental models or related autoimmune and inflammatory conditions, with limited direct validation in patients with SARD-ILD. These proposed interactions may contribute to a self-reinforcing cycle of oxidative stress and Treg dysregulation, potentially promoting effector T-cell activation, fibroblast proliferation, and imbalanced inflammation and repair, thereby contributing to pulmonary fibrotic remodeling. This review integrates current evidence on the potential links among oxidative stress, Treg dysregulation, and pulmonary fibrosis in SARD-ILD, and proposes an emerging mechanistic framework to guide further investigation into their possible interactions and therapeutic implications.

## 1 Introduction

Interstitial lung disease (ILD) comprises a heterogeneous group of inflammatory and fibrotic disorders that primarily affect the pulmonary mesenchyme and represents a major cause of morbidity and mortality in patients with systemic autoimmune rheumatic diseases (SARDs) (1–3). SARD-ILD may occur in rheumatoid arthritis (RA), systemic sclerosis (SSc), microscopic polyangiitis (MPA), Sjögren’s disease (SjD), idiopathic inflammatory myopathies (IIM)-including polymyositis, dermatomyositis, antisynthetase syndrome, and immune-mediated necrotizing myopathy-and mixed connective tissue disease (MCTD) (4, 5). Notably, patients with SSc exhibit a high mortality rate, with a median survival of only 2–3 years in certain cases, thereby imposing a substantial burden on society and families (6). The conventional perspectives attribute pulmonary fibrosis primarily to dysregulated fibroblast activation (7). Accordingly, current clinical treatments primarily aim to inhibit fibroblast activation; however, this approach has shown limited efficacy, serving mainly to delay disease progression rather than reversing fibrosis, with some patients even developing resistance to treatment (8). Recent studies have implicated immune-microenvironment imbalance as a potential contributor to the development of SARD-ILD (9–11). Regulatory T (Treg) cells are essential for maintaining immune tolerance and homeostasis (12), and their functional impairment may be associated with the progression of pulmonary fibrosis. Furthermore, oxidative stress has been implicated in pulmonary fibrotic remodeling. Excessive ROS and RNS may damage cellular macromolecules and promote alveolar epithelial cell apoptosis, macrophage polarization, myofibroblast differentiation, and extracellular-matrix remodeling, thereby contributing to lung injury, inflammation, and fibrosis (13, 14). However, much of this evidence derives from IPF and experimental pulmonary fibrosis models, and its direct relevance to human SARD-ILD remains to be established. Treg homeostasis is also sensitive to metabolic and redox perturbations: ACC1-regulated mitochondrial metabolism influences Treg suppressive function (15), whereas ROS-dependent SOCS3 upregulation can destabilize Tregs in experimental autoimmune settings (16). Taken together, oxidative stress may influence Treg stability through redox-sensitive regulation of Foxp3-associated pathways (16, 17). In parallel, pro-inflammatory macrophage responses may remodel the immune microenvironment (18), whereas context-dependent metabolic cues may modulate Treg homeostasis and suppressive function (19, 20). Collectively, these interacting processes may form a self-reinforcing pathological cycle (16, 21) that disrupts immune homeostasis, promotes excessive effector T cells activation (22, 23), abnormal proliferation of fibroblasts (24), and imbalanced macrophage-mediated inflammation and repair (25), thereby contributing to the progression of pulmonary fibrosis. Given this framework, an in-depth exploration of the molecular mechanisms by which oxidative stress disrupts local pulmonary immune tolerance, alongside a thorough elucidation of the Treg dysfunction in SARD-ILD, holds promise for identifying novel precision therapeutic targets. This narrative review summarizes and critically discusses relevant research advances, with particular attention to the strength, disease context, and translational relevance of the available evidence. Importantly, the collected evidence supporting the relationship among oxidative stress, Treg dysregulation, and pulmonary fibrosis is heterogeneous. Additionally, direct mechanistic evidence from patients with SARD-ILD, particularly from lung tissue or bronchoalveolar lavage fluid, remains limited. Since the majority of the mechanistic insights discussed in this review are derived from experimental pulmonary fibrosis models, other autoimmune diseases, or non-pulmonary inflammatory conditions, the findings of this review are considered supportive or hypothesis-generating evidence rather than direct proof of a causal pathway in human SARD-ILD. To maintain conceptual clarity and scientific rigor, we have distinguished direct SARD-ILD evidence from evidence extrapolated from related experimental and disease settings whenever possible. Accordingly, the proposed oxidative stress-Treg framework should be regarded as a potentially convergent but subtype-dependent hypothesis rather than as a unified mechanism established across RA-ILD, SSc-ILD, MPA-ILD, SjD-ILD, IIM-ILD, and MCTD-ILD.

## 2 Treg dysregulation in SARD-ILD

Tregs are CD4^+^CD25^+^ T cells that play an important role in maintaining immune tolerance and homeostasis, and are characterized by the expression of the transcription factor Foxp3. They regulate immune responses through multiple mechanisms, including interactions with other immune cells and the secretion of regulatory cytokines such as IL-10, IL-35, and TGF-β1 (11, 26). In this review, Treg dysfunction is not inferred from changes in cell frequency alone. We distinguish four related but non-equivalent dimensions: numerical changes in Treg abundance; alterations in regulatory phenotype or molecular programs, including CD25/CTLA-4-associated regulation and IL-10/IL-35 production; loss of lineage stability, such as unstable Foxp3 expression or acquisition of effector-like features; and impaired suppressive capacity demonstrated by functional assays. The term “Treg dysfunction” is used most strictly for loss of lineage stability or suppressive capacity, whereas changes in Treg abundance, Foxp3 expression, or cytokine levels alone are treated as associated phenotypic observations unless supported by complementary functional evidence (12, 26). Treg abnormalities have been increasingly investigated in SARD-ILD, yet the available evidence remains notably heterogeneous, including findings from patients with SARD-ILD, other ILD populations, related autoimmune diseases, and experimental models. For example, studies in patients with fibrotic ILD, including IPF and SARD-ILD, have shown that hilar lymphadenopathy is associated with poorer survival (27). Although these findings suggest that abnormal adaptive immune responses within lung-draining lymph nodes may be associated with disease progression, whether Treg dysregulation directly contributes to these changes remains unclear (27). Collectively, the current evidence supports a possible involvement of Treg dysregulation in SARD-ILD, yet the underlying mechanism remains to be fully established.

### 2.1 Treg phenotypic instability and altered Foxp3 expression

One important aspect of Treg dysregulation is the alteration of Treg phenotype and lineage stability. Foxp3 is a key transcriptional regulator of Treg development, maintenance, and suppressive function (28). Reduced Foxp3 expression may be associated with impaired Treg stability and regulatory capacity, although changes in Foxp3 expression alone do not necessarily demonstrate loss of suppressive function. Recent studies have demonstrated that the transcription factor BATF is essential for maintaining Foxp3 stability. BATF has been identified as an important regulator of Foxp3 stability and Treg lineage maintenance, acting in part through its interaction with the CNS2 region of the Foxp3 locus (29). In mice with Treg-specific BATF deficiency, “ex-Treg” cells with Th17/effector T-cell-like characteristics have been observed, and the lungs are among the organs showing prominent immune-cell infiltration and tissue damage (30). These findings suggest that loss of Treg lineage stability may be accompanied by reduced regulatory characteristics and acquisition of effector-like features, with potential consequences for pulmonary immune homeostasis in experimental settings. However, they do not establish that the same BATF–Foxp3 mechanism operates in human SARD-ILD. Foxp3 expression and lineage stability are also coordinated by multiple regulatory elements within the Foxp3 locus, including CNS0, CNS2, and CNS3, which have distinct but complementary roles in Treg development and maintenance (31). Beyond their intrinsic regulation, Tregs also contribute to the control of excessive effector T-cell responses, including Th1-type inflammation (32, 33). Impaired Treg regulation may therefore favor increased Th1 activity and IFN-γ production, potentially contributing to persistent inflammatory tissue injury. Various mechanisms under different pathological conditions further influence Treg stability. For example, studies in RA have suggested that IL-18 signaling may enhance Foxp3 ubiquitination and degradation, potentially contributing to altered Treg regulation (34). While this finding provides a possible mechanistic clue relevant to autoimmune inflammation, its direct relevance to pulmonary disease remains to be established. Similarly, experimental evidence suggests that loss of IKZF1 function can disrupt the stability of the Foxp3-associated regulatory complex, leading to Treg instability and increased IFN-γ production (35). These findings provide additional evidence that disruption of Treg stability may contribute to dysregulated inflammatory responses, but their specific relevance to SARD-ILD requires further investigation. Taken together, these findings suggest a possible relationship between impaired Treg lineage stability, altered inflammatory responses, and tissue remodeling. Nevertheless, the available evidence does not yet establish a definitive causal sequence from reduced Foxp3 expression to pulmonary fibrosis in human SARD-ILD. Accordingly, the pathway illustrated in **Figure 1** should be interpreted as a proposed mechanistic framework that integrates evidence from diverse experimental and disease settings rather than as a confirmed causal cascade specific to SARD-ILD (**Figure 1**).

**Figure 1:**
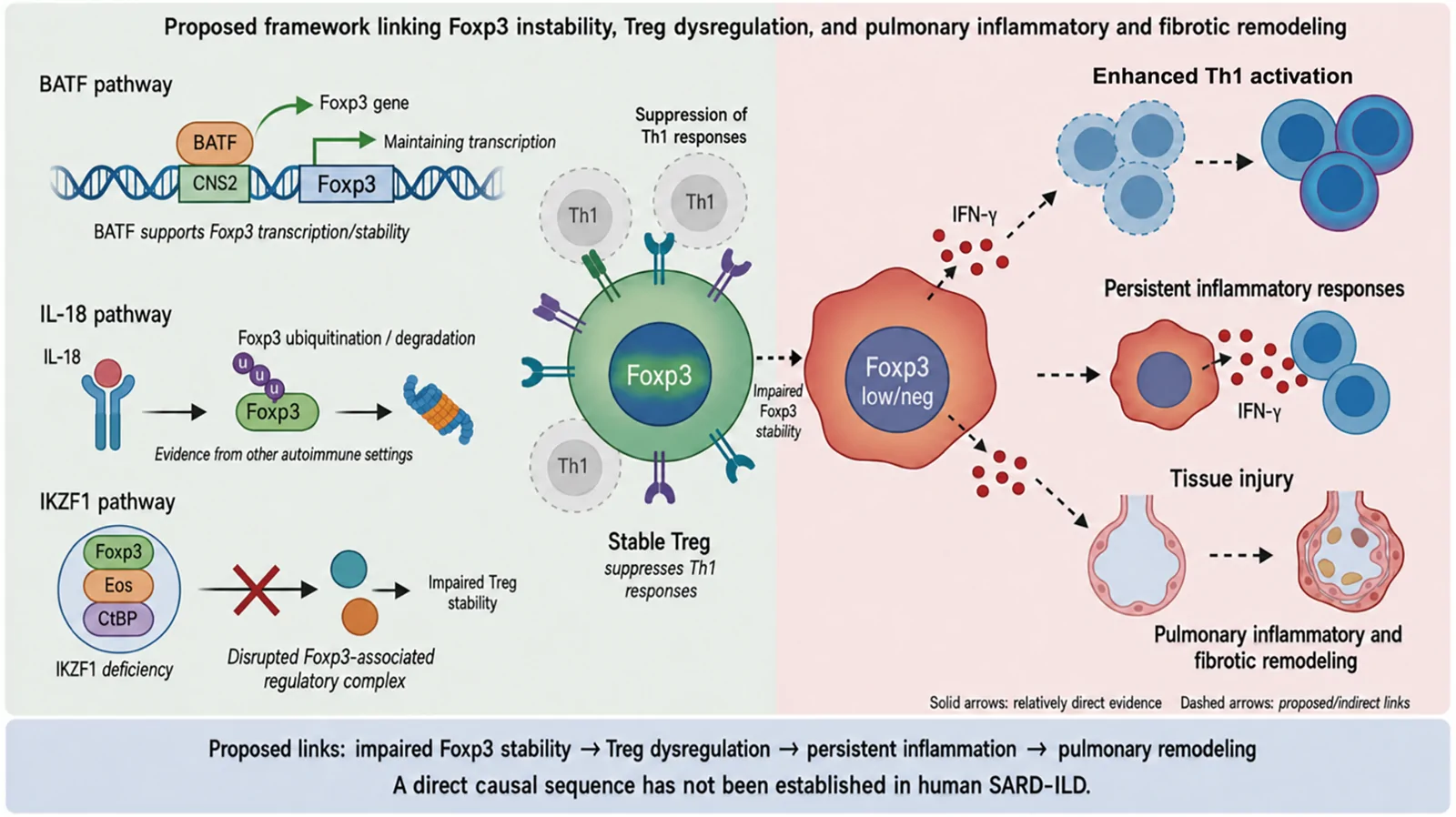
Proposed framework linking Foxp3 instability, Treg dysregulation, and pulmonary inflammatory and fibrotic remodeling. BATF supports Foxp3 transcription and Treg lineage stability through the CNS2 enhancer. Evidence from other autoimmune and inflammatory settings suggests that IL-18 signaling may promote Foxp3 ubiquitination and proteasomal degradation, whereas experimental IKZF1 deficiency disrupts the Foxp3-associated regulatory complex involving Eos and CtBP. These alterations may impair Foxp3 stability and contribute to the emergence of Foxp3-low/negative dysfunctional or ex-Treg-like cells. Stable Tregs normally restrain Th1 responses, whereas impaired Treg stability and regulatory capacity may favor enhanced Th1 activation and IFN-γ production, thereby sustaining inflammatory responses and tissue injury and potentially contributing to pulmonary inflammatory and fibrotic remodeling. Solid arrows indicate relationships supported by relatively direct evidence, whereas dashed arrows indicate proposed or indirect links. This framework integrates evidence from different experimental and disease settings; a direct causal sequence from Foxp3 instability to pulmonary fibrosis has not been established in human SARD-ILD. Created in BioRender. BATF, basic leucine zipper ATF-like transcription factor; CNS2, conserved non-coding sequence 2; CtBP, C-terminal-binding protein; Eos, Ikaros family zinc finger protein 4 (IKZF4); Foxp3, forkhead box P3; IFN-γ, interferon-γ; IKZF1, Ikaros family zinc finger protein 1; SARD-ILD, systemic autoimmune rheumatic disease-associated interstitial lung disease; Th1, T helper 1 cell; Treg, regulatory T cell.

### 2.2 Context-dependent roles of Tregs in pulmonary fibrosis

Current evidence suggests that Treg dysregulation may be associated with pulmonary fibrotic remodeling, although the direction and magnitude of these effects vary across diseases and experimental settings. Experimental studies indicate that changes in Treg abundance can influence pulmonary inflammation and fibrotic remodeling. In the Fra-2 transgenic mouse model, impaired Treg homeostasis was associated with immune imbalance, pulmonary fibrosis, and vascular remodeling, while restoration of Treg populations through adoptive transfer or low-dose IL-2 significantly reduced these pulmonary abnormalities (36). These findings support a potential protective role of Tregs in this experimental setting. However, Treg effects may vary according to their functional state, the local tissue environment, and the stage of disease; Treg-mediated tissue repair may also carry profibrotic consequences under certain conditions. The balance between Treg and Th17/IL-17 responses represents one potentially relevant mechanism. In a bleomycin-induced murine model, inhibition of Th17 differentiation was associated with reduced fibrotic responses (37), while in a separate murine model of acute exacerbation of IPF (AE-IPF), IL-17 deficiency was accompanied by attenuated pulmonary inflammation and fibrosis together with enhanced Treg responses (38). These findings suggest a possible association between the Treg–Th17 balance and pulmonary fibrotic inflammation, although they do not establish that Tregs directly suppress fibrosis through inhibition of Th17/IL-17 responses. Tregs may also influence fibroblast activation and proliferation via tissue-repair and growth-related mediators; however, evidence for these effects is mainly derived from experimental models and other fibrotic diseases and should therefore be considered indirect in the context of SARD-ILD. Treg-derived amphiregulin (Areg) may have context-dependent effects on tissue remodeling. While experimental evidence has shown that Areg can promote fibroblast proliferation, Treg-specific Areg deficiency was associated with reduced allograft fibrosis and vasculopathy in chronic cardiac-allograft model (24). These findings suggest that Treg-mediated tissue repair and fibrotic remodeling may be closely interconnected, although the relevance of this mechanism in the context of SARD-ILD remains uncertain. Human studies in IPF further illustrate that Treg abundance and suppressive function are not equivalent. A study evaluating peripheral blood and bronchoalveolar lavage Tregs in patients with IPF reported globally impaired Treg suppressive function, which was associated with disease severity (39). In a separate study, Sema7A-expressing CD4^+^CD25^+^Foxp3^+^ Tregs were increased in patients with progressive IPF and exhibited reduced expression of regulatory genes, including IL-10. In the experimental component of the same study, adoptive transfer of Sema7A^+^ Tregs promoted pulmonary fibrosis and remodeling in a TGF-β1-driven murine model (40). Although IPF is biologically distinct from SARD-ILD, these findings demonstrate that increased Treg abundance does not necessarily indicate preserved suppressive function and suggest that selected Treg phenotypes may acquire pro-remodeling properties in a fibrotic lung environment. Experimental studies further suggest that Treg depletion may affect pulmonary macrophage homeostasis. The heightened susceptibility of Treg-deficient mice to pulmonary fibrosis is closely linked to innate immune dysregulation. In transgenic mouse models, *in vivo* depletion of Tregs results in impaired maturation of monocyte-derived alveolar macrophages (Mo-AMs), characterized by decreased expression of the surface marker SiglecF. This maturation defect hinders the transition of macrophages from a pro-inflammatory to a reparative phenotype, thereby exacerbating disease severity and fibrosis. In these experimental models, Treg-derived IL-10 was identified as an important factor supporting macrophage maturation and limiting fibrotic progression (25). A study incorporating experimental lung injury models further showed that Tregs may promote alveolar epithelial repair through growth factors such as amphiregulin (Areg) and keratinocyte growth factor (KGF), while also limiting fibroproliferative remodeling by reducing the recruitment of bone marrow-derived collagen-producing cells through the CXCL12/CXCR4 axis (21). These findings suggest that Tregs may contribute to the tissue environment that favors repair over fibrosis in some settings, although the balance between their reparative and potentially pro-fibrotic effects may depend on the local microenvironment and disease stage (21). Overall, current evidence supports a potential but context-dependent role for Treg dysregulation in SARD-ILD and pulmonary fibrotic remodeling. Nevertheless, this evidence is derived from heterogeneous sources—including human SARD-ILD studies, other autoimmune or fibrotic diseases, and experimental models—which should not be considered equivalent. Changes in Treg abundance, phenotype, lineage stability, and suppressive function should therefore be interpreted separately. Direct mechanistic evidence from human SARD-ILD remains limited, and further disease- and tissue-specific studies are needed.

## 3 Oxidative stress and Treg dysregulation

Treg metabolism is characterized by substantial reliance on oxidative phosphorylation (OXPHOS) and fatty acid oxidation (FAO), which contribute to the maintenance of their metabolic fitness and suppressive programs (15). Mitochondrial integrity is likewise important for Treg suppressive capacity (15, 41). Under oxidative stress, lipid peroxidation generates reactive products such as 4-hydroxynonenal (4-HNE). Experimental studies in other cellular systems have shown that 4-HNE can modify mitochondrial proteins and that oxidative stress can interfere with mitochondrial dynamics (42–44), suggesting a potential mechanism through which lipid peroxidation may influence Treg metabolic fitness. However, this mechanism has not been directly established in SARD-ILD. Beyond direct metabolic effects, metabolic stress may also influence Treg epigenetic programs. In Treg-specific TKT-deficient experimental models, reduced α-ketoglutarate (α-KG) availability was associated with DNA hypermethylation, reduced chromatin accessibility, and decreased expression of activated-Treg-associated genes, including Il10, Ccr4, and Icos (20). These findings suggest that metabolic changes may influence Treg regulatory programs through epigenetic mechanisms. Lipid composition represents another metabolic dimension that may influence Treg function. In multiple sclerosis, altered lipid availability has been linked to impaired suppressive properties of tissue-resident Tregs, whereas oleic acid supplementation partially restored their regulatory phenotype (41). Although this evidence is extrapolated from multiple sclerosis rather than SARD-ILD, it suggests that local lipid composition may be one metabolic factor capable of influencing Treg homeostasis. Together, these observations suggest that excessive or sustained ROS accumulation may influence Treg homeostasis through interconnected metabolic, mitochondrial, and epigenetic pathways.

ROS, as signaling molecules, modulate a wide range of biological and physiological processes via regulating the activity of various redox-sensitive targets (45, 46). Precise control of intracellular ROS levels is therefore essential for maintaining cellular homeostasis. Excessive ROS accumulation and oxidative stress have been reported in several autoimmune diseases, including RA (47, 48). Elevated ROS levels have been observed in patients with RA and in collagen-induced arthritis (CIA) mouse models (16). In experimental T-cell cultures, hydrogen peroxide exposure was associated with a reduction in Treg frequency and Foxp3 expression (16), suggesting that Tregs may be particularly sensitive to excessive oxidative stress. However, these findings derive from RA/CIA-related experimental settings and therefore provide indirect rather than direct evidence for SARD-ILD. Current evidence suggests that oxidative stress may influence Treg homeostasis through several interconnected processes, including redox-sensitive regulation of Foxp3 expression and stability, modulation of IL-2/STAT5 signaling, metabolic reprogramming, and remodeling of the inflammatory microenvironment. Importantly, these mechanisms are supported by evidence of varying strength and have not all been directly demonstrated in human SARD-ILD. The underlying mechanisms are discussed below from three perspectives.

### 3.1 Oxidative stress-mediated regulation of Foxp3 expression and stability

Foxp3 expression and protein stability are central to the maintenance of Treg lineage identity. Recent experimental studies further indicate that the requirement for Foxp3 can vary with inflammatory context, highlighting the importance of maintaining Foxp3 expression during sustained immune activation (49). In inflammatory environments, the stability of the Foxp3 protein is tightly regulated by various post-translational modifications. For example, in obesity-associated inflammatory settings, IL-18 signaling was shown to be associated with STUB1-mediated ubiquitination and proteasomal degradation of Foxp3 (34); however, whether this mechanism operates in SARD-ILD remains unknown. In an experimental model of intestinal inflammation, succinate accumulation was shown to alter Foxp3 succinylation and increase its susceptibility to ubiquitination and degradation (50), providing additional extrapolated evidence for metabolic regulation of Foxp3 stability. Post-translational mechanisms also contribute to Foxp3 stability. USP1 has been shown to indirectly promote Foxp3 proteasomal degradation by deubiquitinating and stabilizing TAZ, thereby favoring Th17-cell differentiation and attenuating Treg-cell differentiation (51). In contrast, activated AMPK can directly phosphorylate Foxp3, reducing its ubiquitination and proteasomal degradation and thereby maintaining Foxp3 protein stability (52). These findings illustrate opposing post-translational mechanisms that regulate Foxp3 stability; however, their direct relevance to SARD-ILD remains to be established. Collectively, these studies indicate that Foxp3 stability can be regulated by multiple post-translational mechanisms, including ubiquitination, succinylation, and phosphorylation, yet, much of this evidence derives from non-SARD inflammatory settings. Consequently, the direct contribution of these regulatory mechanisms to Treg dysregulation in SARD-ILD remains uncertain.

Oxidative stress may influence Foxp3 stability through redox-sensitive metabolic and signaling pathways. For example, ROS-associated HIF-1α signaling has been implicated in the regulation of Foxp3 stability under metabolically stressed conditions (17). In addition, previous studies have demonstrated that Tregs express phosphatase and tensin homologue (PTEN) at higher levels than other CD4^+^ T cell subsets (53), indicating that PTEN is a key regulator of Treg stability (54, 55). Increased expression of NADPH oxidase 2 (Nox2) in Tregs has been shown to promote ROS accumulation. In experimental models of autoimmune disease, ROS were shown to induce oxidative modification and inactivation of PTEN in Tregs, resulting in activation of the downstream Akt/mTOR/STAT3 pathway and subsequent suppressor of cytokine signaling 3 (SOCS3) upregulation (16). Increased SOCS3 expression was associated with reduced Foxp3 expression and impaired Treg stability. These findings support the ROS-PTEN-Akt/mTOR/STAT3-SOCS3 axis as a potential mechanism linking redox stress to Treg instability; though, this pathway has not yet been directly demonstrated in SARD-ILD. It should be noted that ROS are not uniformly detrimental to T-cell biology. Physiological mitochondrial ROS can function as signaling intermediates during T-cell activation, including the activation of NFAT (56, 57). Separately, CNS2 has been shown to maintain Foxp3 expression and Treg identity by sensing TCR/NFAT signaling and facilitating its interaction with the Foxp3 promoter (58). Taken together, these findings suggest that ROS effects on Treg biology may be context dependent, rather than following a uniformly detrimental or simple linear relationship.

In summary, current evidence suggests that oxidative stress may influence Foxp3 expression and stability through redox-sensitive signaling, metabolic regulation, and transcriptional or post-transcriptional mechanisms (16, 56–60). Foxp3 protein stability is also controlled by post-translational mechanisms, including ubiquitination, proteasomal degradation, and phosphorylation (51, 52). However, a direct causal link between ROS-mediated oxidation of Foxp3 and its subsequent ubiquitination or degradation has not been established. Therefore, direct oxidative modification of Foxp3 should currently be regarded as an unconfirmed mechanism rather than an established pathway in SARD-ILD (**Figure 2**).

**Figure 2:**
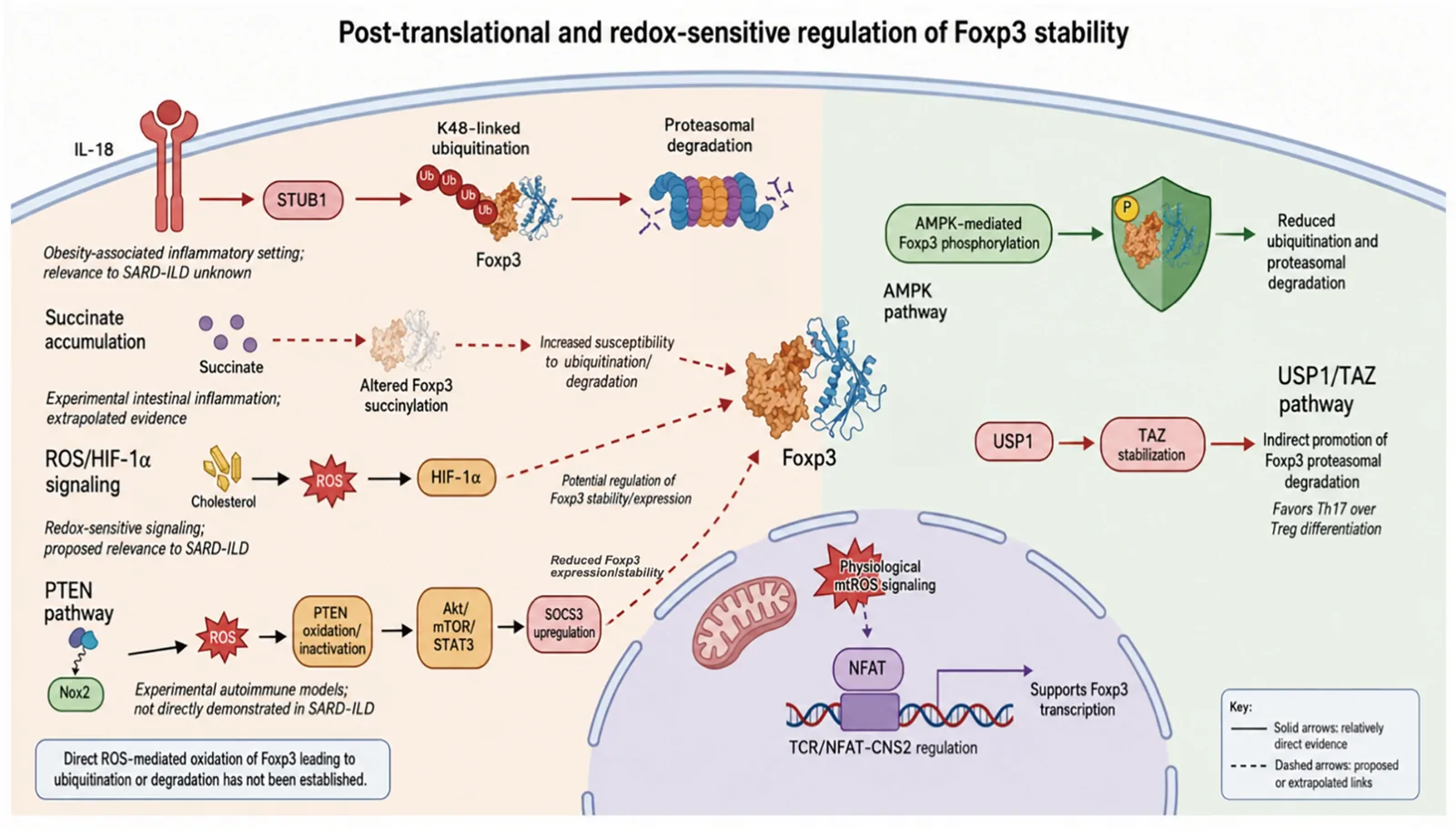
Post-translational and redox-sensitive regulation of Foxp3 stability. Foxp3 expression and protein stability are regulated by several post-translational, metabolic, and redox-sensitive mechanisms supported by evidence of varying strength. In an obesity-associated inflammatory setting, IL-18 signaling has been associated with STUB1-mediated K48-linked ubiquitination and proteasomal degradation of Foxp3, although the relevance of this pathway to SARD-ILD remains unknown. In experimental intestinal inflammation, succinate accumulation alters Foxp3 succinylation and increases its susceptibility to ubiquitination and degradation, providing extrapolated evidence for metabolic regulation of Foxp3 stability. Cholesterol-associated ROS/HIF-1α signaling may also influence Foxp3 expression or stability, whereas, in experimental autoimmune models, Nox2-derived ROS oxidize and inactivate PTEN, activating the Akt/mTOR/STAT3–SOCS3 pathway and reducing Foxp3 expression and Treg stability; neither pathway has been directly demonstrated in SARD-ILD. In contrast, AMPK-mediated phosphorylation of Foxp3 reduces its ubiquitination and proteasomal degradation, thereby preserving Foxp3 stability. USP1 indirectly promotes Foxp3 proteasomal degradation by stabilizing TAZ, favoring Th17-cell differentiation over Treg-cell differentiation. Physiological mitochondrial ROS may function as signaling intermediates that activate NFAT, while TCR/NFAT–CNS2 regulation supports Foxp3 transcription; however, this integrated link remains indirect. Importantly, a direct causal mechanism in which ROS-mediated oxidation of Foxp3 leads to its ubiquitination or degradation has not been established. Solid arrows indicate relationships supported by relatively direct evidence, whereas dashed arrows represent proposed, indirect, or extrapolated links. Created in BioRender. Akt, protein kinase B; AMPK, AMP-activated protein kinase; CNS2, conserved non-coding sequence 2; Foxp3, forkhead box P3; HIF-1α, hypoxia-inducible factor 1α; K48, lysine 48; mTOR, mechanistic target of rapamycin; mtROS, mitochondrial reactive oxygen species; NFAT, nuclear factor of activated T cells; Nox2, NADPH oxidase 2; PTEN, phosphatase and tensin homolog; ROS, reactive oxygen species; SARD-ILD, systemic autoimmune rheumatic disease-associated interstitial lung disease; SOCS3, suppressor of cytokine signaling 3; STAT3, signal transducer and activator of transcription 3; STUB1, STIP1 homology and U-box-containing protein 1; TAZ, transcriptional coactivator with PDZ-binding motif; TCR, T-cell receptor; Treg, regulatory T cell; USP1, ubiquitin-specific protease 1.

### 3.2 Oxidative stress and IL-2/STAT5 signaling

IL-2/STAT5 signaling is essential for Treg development, maintenance, and regulatory function, and experimental evidence suggests that oxidative stress may interfere with components of this pathway. Activated STAT5 directly binds the distal CNS0 enhancer within the Foxp3 locus, thereby supporting IL-2-dependent Foxp3 induction and thymic Treg lineage commitment (61). However, this study establishes the importance of the IL-2/STAT5-CNS0 axis in Treg development without addressing whether oxidative stress modulates this pathway; thus, the contribution of redox stress to IL-2/STAT5 signaling in SARD-ILD remains uncertain. Evidence from experimental immune-regulatory systems indicates that alterations in CD25 availability or gp96 function can reduce STAT5 phosphorylation and disturb IL-2-dependent Treg homeostasis (62, 63). These findings illustrate potential regulatory points within the IL-2/STAT5 pathway, although their relevance to SARD-ILD remains to be established (62, 63). In multiple sclerosis, peroxynitrite (ONOO^−^)-mediated nitration of the IL-2 receptor has been reported to interfere with IL-2 signaling and downstream pathways (64). While this finding provides evidence that reactive nitrogen species can affect IL-2 receptor signaling in an autoimmune setting, its relevance to SARD-ILD remains extrapolative and requires direct validation. Collectively, these studies suggest that redox and metabolic disturbances may influence IL-2/STAT5-dependent Treg regulation through several upstream mechanisms. However, most of the available evidence is derived from experimental systems or autoimmune diseases other than SARD-ILD, and direct evidence for oxidative disruption of IL-2/STAT5 signaling in human SARD-ILD remains limited (**Figure 3**).

**Figure 3:**
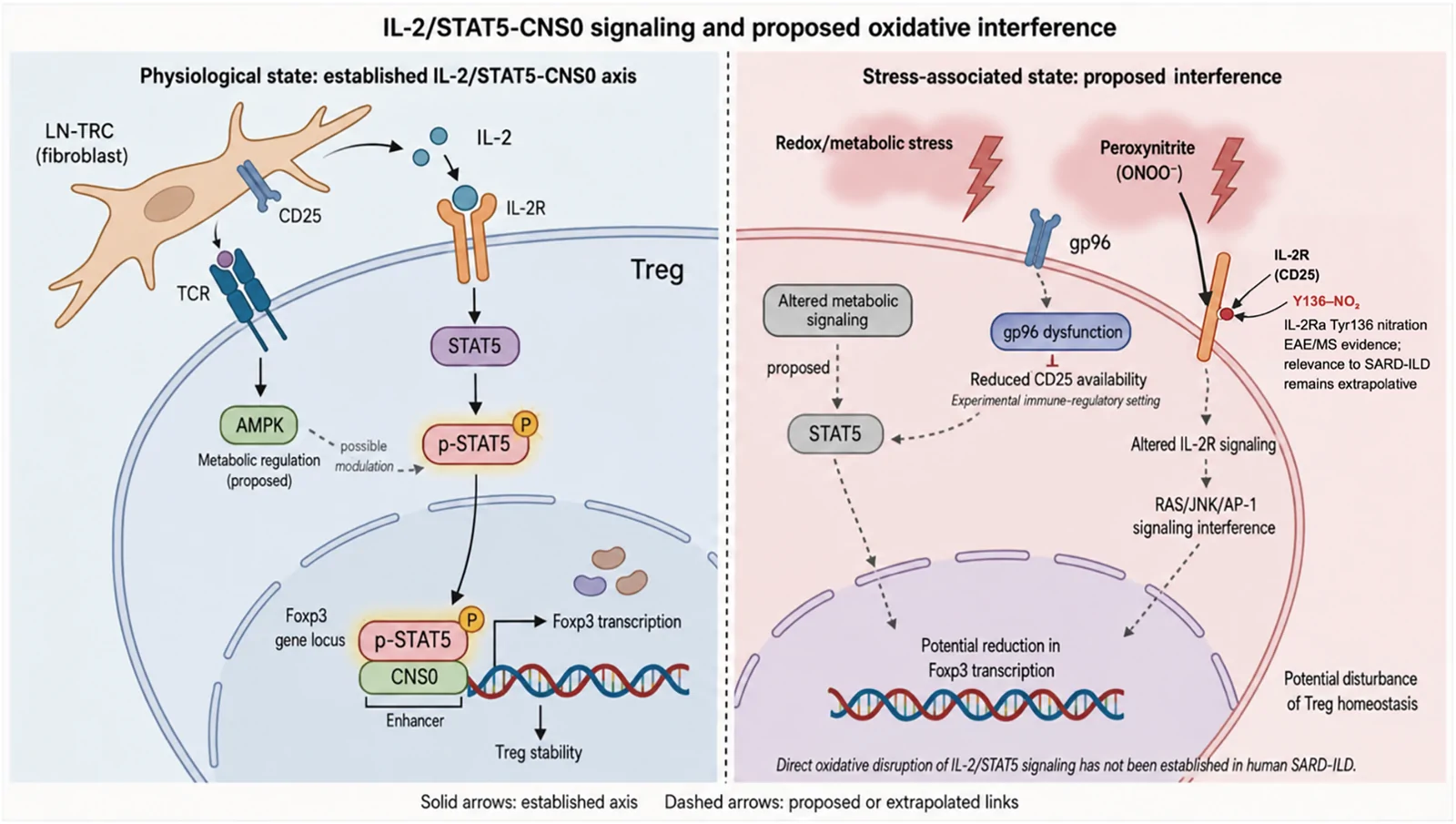
IL-2/STAT5–CNS0 signaling and proposed oxidative interference. Under physiological conditions, lymph node T-zone reticular cells and CD25-dependent IL-2 availability support IL-2 receptor signaling in Tregs. IL-2 binding to IL-2R promotes STAT5 phosphorylation, after which phosphorylated STAT5 binds the distal CNS0 enhancer within the Foxp3 locus, supporting Foxp3 transcription and Treg lineage stability. AMPK-mediated metabolic regulation is depicted as a possible modulator of STAT5 phosphorylation, although this relationship remains proposed. Under stress-associated conditions, redox or metabolic disturbances may interfere with the IL-2/STAT5 pathway at several upstream regulatory points. Experimental immune-regulatory studies indicate that gp96 dysfunction and reduced CD25 availability can impair STAT5 activation and disturb IL-2-dependent Treg homeostasis. In experimental autoimmune encephalomyelitis and multiple sclerosis, peroxynitrite-mediated nitration of Tyr136 within IL-2Rα/CD25 has been associated with altered IL-2R signaling and interference with downstream RAS/JNK/AP-1 signaling. These disturbances may reduce STAT5-dependent Foxp3 transcription and potentially impair Treg homeostasis. However, evidence for these stress-associated mechanisms is derived primarily from experimental systems or autoimmune diseases other than SARD-ILD, and direct oxidative disruption of IL-2/STAT5 signaling has not been established in human SARD-ILD. Solid arrows indicate the established IL-2/STAT5–CNS0 axis, whereas dashed arrows represent proposed, indirect, or extrapolated links. Created in BioRender. AMPK, AMP-activated protein kinase; AP-1, activator protein 1; CD25, IL-2 receptor α-chain; CNS0, conserved non-coding sequence 0; EAE, experimental autoimmune encephalomyelitis; Foxp3, forkhead box P3; gp96, glycoprotein 96; IL-2, interleukin-2; IL-2R, interleukin-2 receptor; JNK, c-Jun N-terminal kinase; LN-TRC, lymph node T-zone reticular cell; MS, multiple sclerosis; ONOO^−^, peroxynitrite; p-STAT5, phosphorylated signal transducer and activator of transcription 5; RAS, rat sarcoma GTPase; SARD-ILD, systemic autoimmune rheumatic disease-associated interstitial lung disease; TCR, T-cell receptor; Treg, regulatory T cell; Tyr136, tyrosine residue 136.

### 3.3 ROS-induced microenvironmental remodeling

Mitochondrial damage-associated ROS can promote NLRP3 inflammasome activation in macrophages, leading to caspase-1-dependent IL-1β release. In an osimertinib-treated tumor model, this inflammatory response was accompanied by a reduction in Tregs within the tumor microenvironment, although a direct causal effect of IL-1β on Treg differentiation or function was not established (65). Importantly, NLRP3 inflammasome activation is not restricted to innate immune cells. Tfh cells can also exhibit NLRP3/caspase-1 activation and produce IL-1β. In SLE, expansion of NLRP3-activated Tfh cells correlated with disease activity, suggesting that inflammasome signaling may also contribute to adaptive immune dysregulation in systemic autoimmunity (66).

Macrophage polarization represents another potential interface between the immune microenvironment and Treg homeostasis. In a murine model of chronic social stress, increased oxytocin (OT) signaling was associated with expansion of Foxp3^+^ Tregs and polarization of macrophages toward an M2-like phenotype, together with reduced pro-inflammatory and increased anti-inflammatory cytokine responses (67). Conversely, pharmacological blockade of OTR with L-371257 reduced Foxp3^+^ Tregs, increased M1-like macrophages, decreased M2-like macrophages, and shifted the cytokine profile toward a more pro-inflammatory state, characterized by increased IL-1β and IFN-γ and decreased IL-4 and TGF-β. A similar immunological shift was also observed after splenic denervation (67). In a murine lung cancer model, proton immunoradiotherapy, particularly NBTXR3 combined with proton radiotherapy and anti-PD-1 treatment, was associated with enhanced M1-like macrophage polarization, increased TNF-α expression, suppressed TGF-β-related signaling, and a decreased proportion of Tregs compared with the corresponding photon-based regimen (68). These findings illustrate the close association between macrophage polarization, cytokine composition, and Treg abundance, although the direction and functional consequences of these relationships are likely to depend on the disease context. Importantly, macrophage polarization and oxidative stress may also interact bidirectionally. In tumor models, repolarization of tumor-associated macrophages toward an M1-like phenotype was shown to increase TNF-α expression and ROS production. ROS generated during this process, in turn, reduced IL-2 production by activated CD4^+^ T cells and increased apoptosis of CD4^+^CD25^+^Foxp3^+^ Tregs, whereas catalase-mediated removal of excess ROS attenuated Treg apoptosis (69). These observations support a mechanism by which macrophage-derived oxidative stress can reshape local T-cell responses, although its relevance to SARD-ILD remains to be established.

Beyond microenvironmental effects, excessive mitochondrial ROS can also directly impair chronically activated T cells. Recent studies have shown that mitochondrial ROS induces telomeric DNA damage and telomere fragility, triggering a DNA damage response involving ATR–CHK1 signaling, p53 upregulation, and downstream MDM2/p21 expression, accompanied by reduced proliferation and cytokine production and increased expression of exhaustion-associated markers (70, 71). Although persistent T-cell activation in autoimmune disease may create similar oxidative pressure, whether ROS-mediated telomere damage directly contributes to Treg dysfunction in SARD-ILD remains to be determined.

Collectively, these findings indicate that oxidative stress can influence T-cell and Treg homeostasis through both cell-extrinsic mechanisms—including inflammasome activation and macrophage-associated microenvironmental remodeling—and cell-intrinsic mechanisms, such as disruption of IL-2 signaling and oxidative damage to telomeres. However, as several of these mechanisms have been defined primarily in tumor or other experimental models, their direct contribution to Treg dysfunction in SARD-ILD requires further validation (**Figure 4**).

**Figure 4:**
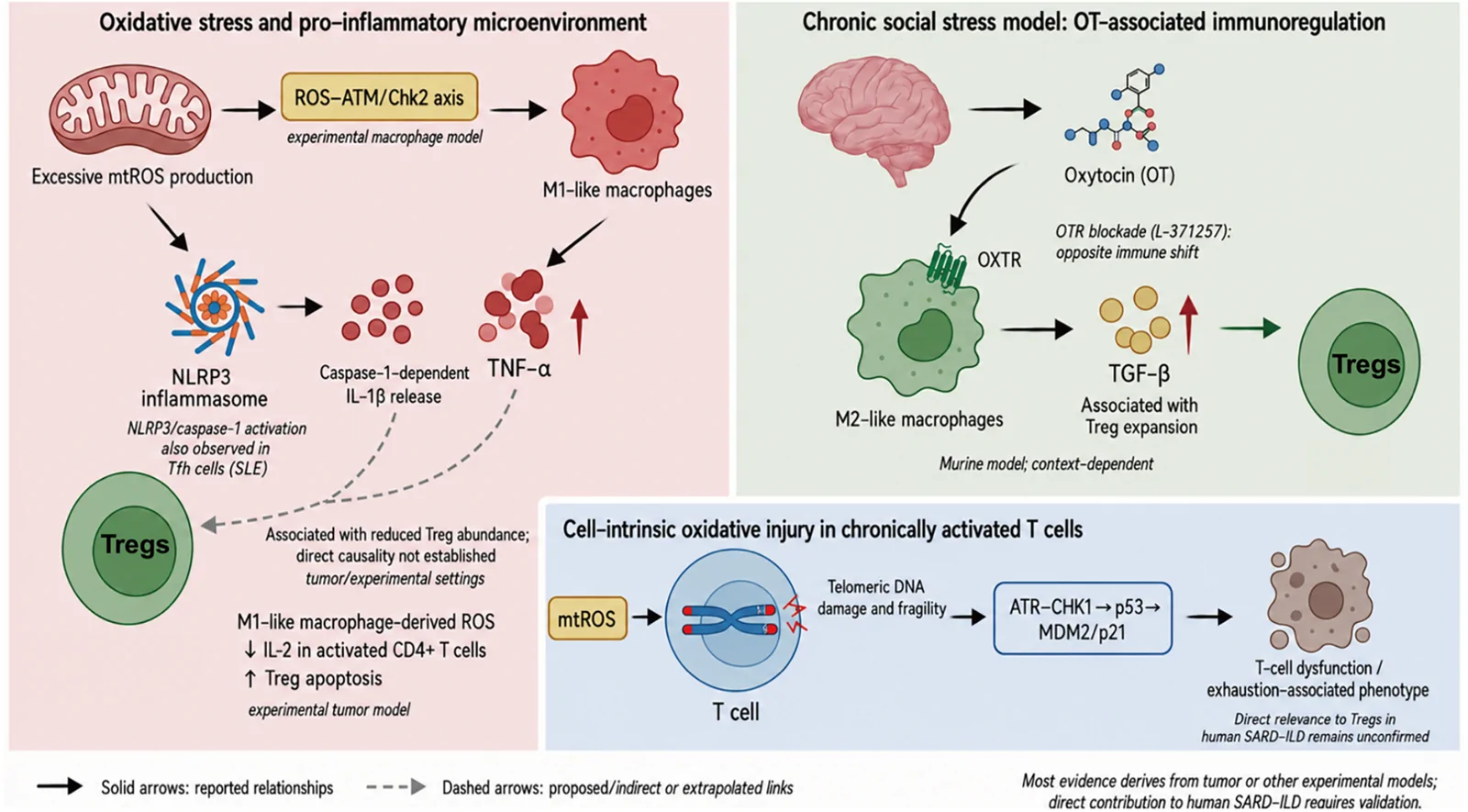
Proposed mechanisms linking oxidative stress, inflammatory microenvironment remodeling, and T-cell/Treg dysfunction. Excessive mitochondrial ROS may remodel the immune microenvironment through several cell-extrinsic and cell-intrinsic mechanisms. In experimental macrophage and tumor models, mitochondrial ROS promote NLRP3 inflammasome activation and caspase-1-dependent IL-1β release, while the ROS–ATM/Chk2 axis is associated with M1-like macrophage polarization and increased TNF-α production. NLRP3/caspase-1 activation and IL-1β production have also been observed in Tfh cells in SLE. These inflammatory changes have been associated with reduced Treg abundance in experimental settings, although a direct causal effect of IL-1β or TNF-α on Treg differentiation or function has not been established. In addition, M1-like macrophage-derived ROS can reduce IL-2 production by activated CD4^+^ T cells and increase Treg apoptosis in experimental tumor models. In a murine model of chronic social stress, oxytocin signaling through OXTR was associated with M2-like macrophage polarization, increased TGF-β production, and Foxp3^+^ Treg expansion, whereas pharmacological OXTR blockade with L-371257 produced an opposite immune shift; these effects are context-dependent. At the cell-intrinsic level, mitochondrial ROS can induce telomeric DNA damage and fragility in chronically activated T cells, activating the ATR-CHK1-p53-MDM2/p21 response and contributing to reduced proliferation, impaired cytokine production, and an exhaustion-associated phenotype. Whether this telomere-damage pathway directly contributes to Treg dysfunction in human SARD-ILD remains unconfirmed. Solid arrows indicate reported relationships, whereas dashed arrows represent proposed, indirect, or extrapolated links. Most supporting evidence derives from tumor or other experimental models, and the direct contribution of these mechanisms to human SARD-ILD requires validation. Created in BioRender. ATM, ataxia telangiectasia mutated; ATR, ataxia telangiectasia and Rad3-related protein; CHK1/Chk2, checkpoint kinase 1/2; Foxp3, forkhead box P3; IL-1β, interleukin-1β; IL-2, interleukin-2; MDM2, mouse double minute 2 homolog; mtROS, mitochondrial reactive oxygen species; NLRP3, NLR family pyrin domain-containing 3; OT, oxytocin; OXTR, oxytocin receptor; ROS, reactive oxygen species; SARD-ILD, systemic autoimmune rheumatic disease-associated interstitial lung disease; SLE, systemic lupus erythematosus; Tfh, follicular helper T cell; TGF-β, transforming growth factor-β; TNF-α, tumor necrosis factor-α; Treg, regulatory T cell.

## 4 Context-dependent links between Treg dysregulation and pulmonary fibrosis

The balance between Tregs and Th17 responses is an important component of immune homeostasis, and its disruption has been consistently associated with enhanced Th17-mediated inflammation in experimental and autoimmune settings (22, 23). IL-17-mediated inflammation is thought to contribute to pulmonary fibrotic remodeling in the lung. In an experimental AE-IPF model, IL-17 deficiency resulted in reduced neutrophil and eosinophil responses together with attenuated pulmonary inflammation and fibrosis (38). Importantly, γδ T cells were the predominant source of IL-17 in this model, indicating that these findings should not be interpreted as evidence for a Th17-specific pathogenic mechanism. Beyond IL-17, evidence from non-pulmonary fibrotic disease suggests that Th17 cells may also influence fibrosis through alternate mechanisms independent of IL-17. In experimental colitis and Crohn’s disease, for instance, Th17-derived amphiregulin (Areg) was shown to promote myofibroblast proliferation, migration, and collagen I expression through mTOR- and MEK-dependent signaling cascades (72). Nevertheless, whether a similar Th17–Areg–mTOR/MEK pathway contributes to pulmonary fibrosis or SARD-ILD remains unknown. Independent evidence from pulmonary fibrosis models has identified RUNX2 as a key regulator of fibroblast fate. In bleomycin-induced pulmonary fibrosis, RUNX2 was found to be enriched in pathological fibroblasts, and fibroblast-specific Runx2 deletion significantly reduced the transition of alveolar fibroblasts into pathological fibroblasts, attenuated extracellular matrix deposition, and decreased pulmonary fibrosis (73). Increased RUNX2 expression was also observed in pathological fibroblasts from human IPF lungs (73). While TGF-β signaling is known to be associated with fibrotic fibroblast programs, the available evidence does not establish a direct mechanistic link among Th17 cells, TGF-β, and RUNX2 in this context. Beyond Th17 cells, Tregs themselves may also exert context-dependent effects on tissue remodeling. In a murine model of chronic cardiac allograft rejection, Treg-derived Areg promoted fibroblast proliferation, whereas Treg-specific Areg deficiency was associated with reduced allograft fibrosis and vasculopathy (24). These observations suggest that Treg-mediated tissue repair and fibrotic remodeling may overlap and that Tregs should not be considered uniformly antifibrotic (21). Importantly, however, these findings stem primarily from acute lung injury and infection models, and their relevance to the chronic fibrotic process characteristic of SARD-ILD remains to be determined.

Alterations in macrophage phenotype and maturation represent another potential interface between Treg dysregulation and fibrotic remodeling. In experimental pulmonary fibrosis models, Treg-derived IL-10 has been identified as a critical mediator that supports the maturation of monocyte-derived alveolar macrophages (Mo-AMs) toward a homeostatic phenotype while limiting fibrotic progression (25). Additionally, macrophage-derived cytokine profiles have been shown to influence fibroblast activation and extracellular matrix remodeling in these models. In a three-dimensional pulmonary fibrosis system, M1 macrophages were associated with increased IL-6 production, whereas M2 macrophages produced higher levels of IL-4, IL-13, and TGF-β; both macrophage states were linked to fibrotic mechanical remodeling (74).

Evidence from a non-pulmonary fibrotic model provides additional support for Treg–macrophage crosstalk in regulating fibrotic outcomes. In experimental chronic pancreatitis, Treg depletion was found to enhance type-2 immune responses and increased alternatively activated CD206^+^ macrophages, accumulation. This was accompanied by increased macrophage-associated pro-fibrotic growth-factor signaling, including activin A, TGF-β, and FGF1, as well as greater collagen deposition (75). Collectively, these findings suggest that loss of Treg-mediated regulation can promote a macrophage-associated pro-fibrotic environment in some organs; however, whether this same mechanistic axis operates in pulmonary fibrosis or SARD-ILD remains unknown.

Conceptually, preserved Treg-mediated suppression may be more protective during inflammation-dominant phases, whereas persistent or phenotypically altered tissue-repair programs may contribute to remodeling during later fibrotic phases; however, direct longitudinal evidence in human SARD-ILD remains lacking. Taken together, the current evidence does not support a uniformly protective or uniformly pro-fibrotic role for Tregs in pulmonary fibrosis. Preserved Treg-mediated immune regulation may limit excessive inflammatory responses and support macrophage and epithelial homeostasis, whereas Treg-derived repair mediators such as Areg may promote fibroblast proliferation and remodeling under particular conditions. However, several proposed mechanistic links—particularly the Th17-Areg pathway and type-2 Treg-macrophage interactions described in intestinal or pancreatic fibrosis—are derived from non-pulmonary settings and thus remain extrapolative with respect to SARD-ILD. In contrast, experimental lung-fibrosis model studies provide more direct support for Treg-derived IL-10 in regulating alveolar macrophage maturation (25), although confirmation in human SARD-ILD remains lacking. Overall, the net effect of Tregs is likely to depend on disease stage, tissue compartment, local microenvironment, and Treg functional state (**Figure 5**; **Supplementary Table 1**).

**Figure 5:**
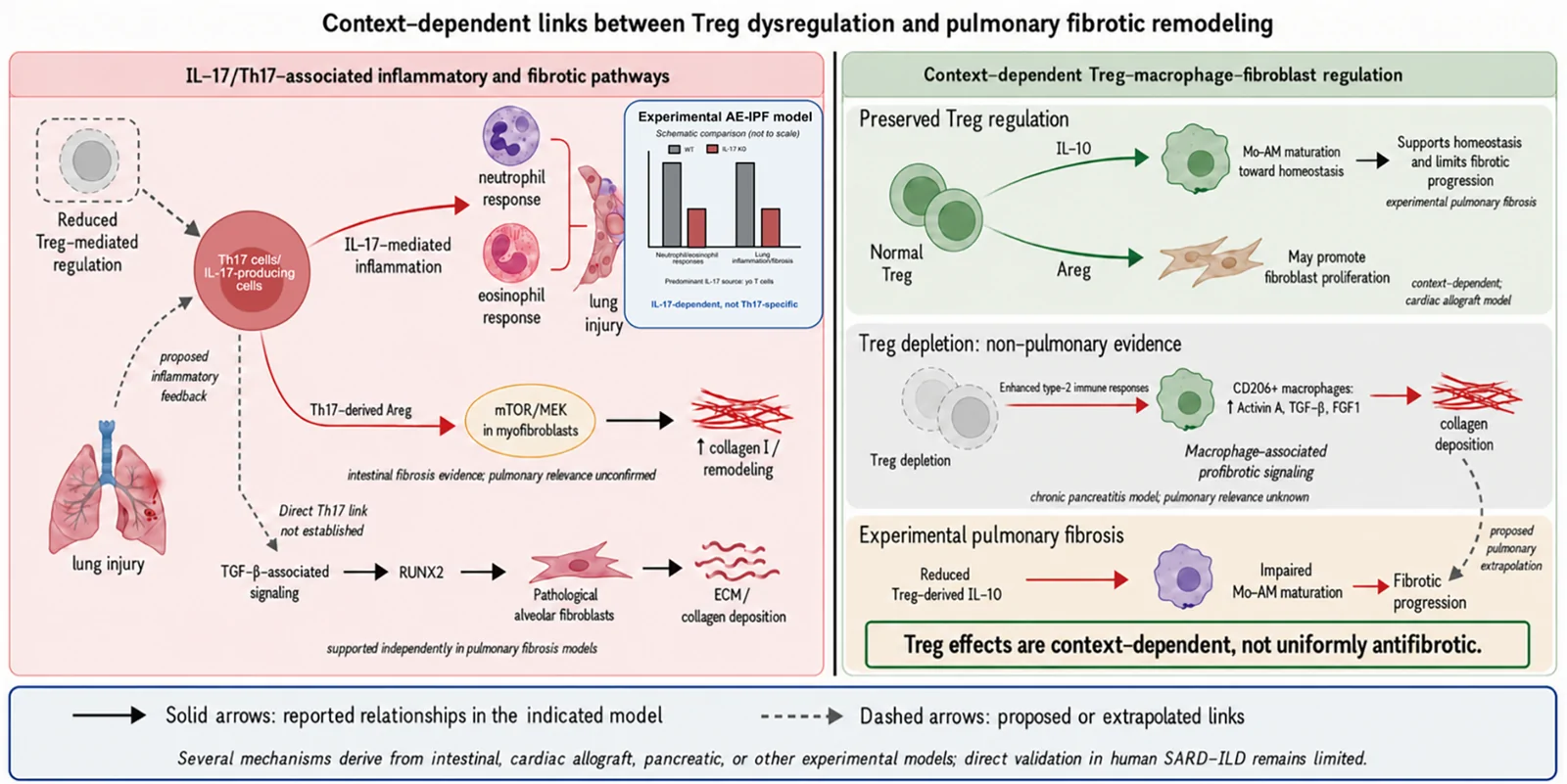
Context-dependent links between Treg dysregulation and pulmonary fibrotic remodeling. Reduced Treg-mediated regulation is proposed to favor Th17 or other IL-17-producing cell responses, although a direct causal relationship has not been established. In an experimental acute exacerbation of idiopathic pulmonary fibrosis model, IL-17 deficiency was associated with reduced neutrophil and eosinophil responses and attenuated pulmonary inflammation and fibrosis. However, γδ T cells were the predominant source of IL-17 in this model; therefore, these findings support an IL-17-dependent rather than a specifically Th17-mediated mechanism. In experimental intestinal fibrosis, Th17-derived amphiregulin promotes myofibroblast activation and collagen I expression through mTOR/MEK signaling, but the pulmonary relevance of this pathway remains unconfirmed. Independently, pulmonary fibrosis models support a role for TGF-β-associated RUNX2 signaling in the transition of alveolar fibroblasts into pathological fibroblasts and subsequent extracellular matrix deposition; a direct Th17-TGF-β-RUNX2 pathway has not been demonstrated. Tregs may also exert context-dependent effects on macrophage and fibroblast biology. In experimental pulmonary fibrosis, Treg-derived IL-10 supports the maturation of monocyte-derived alveolar macrophages toward a homeostatic phenotype and limits fibrotic progression, whereas reduced Treg-derived IL-10 is associated with impaired macrophage maturation and enhanced fibrosis. Conversely, Treg-derived amphiregulin may promote fibroblast proliferation in a chronic cardiac allograft model. In experimental chronic pancreatitis, Treg depletion enhances type-2 immune responses and increases CD206^+^ macrophage-associated profibrotic signaling, including activin A, TGF-β, and FGF1, accompanied by increased collagen deposition; whether this mechanism operates in pulmonary fibrosis remains unknown. Thus, Treg effects are context-dependent and should not be considered uniformly antifibrotic. Solid arrows indicate reported relationships within the specified experimental models, whereas dashed arrows represent proposed or extrapolated links. Several mechanisms derive from intestinal, cardiac allograft, pancreatic, or other experimental models, and their direct relevance to human SARD-ILD requires validation. Created in BioRender. AE-IPF, acute exacerbation of idiopathic pulmonary fibrosis; Areg, amphiregulin; ECM, extracellular matrix; FGF1, fibroblast growth factor 1; IL-10, interleukin-10; IL-17, interleukin-17; MEK, mitogen-activated protein kinase kinase; Mo-AM, monocyte-derived alveolar macrophage; mTOR, mechanistic target of rapamycin; RUNX2, runt-related transcription factor 2; SARD-ILD, systemic autoimmune rheumatic disease-associated interstitial lung disease; TGF-β, transforming growth factor-β; Th17, T helper 17 cell; Treg, regulatory T cell.

## 5 Therapeutic implications of targeting oxidative stress and Treg dysregulation

Current management of SARD-ILD is disease- and phenotype-specific, relying primarily on immunomodulatory therapy, with antifibrotic treatment incorporated in selected fibrosing phenotypes. According to the 2023 ACR/CHEST guideline, mycophenolate is conditionally recommended as a preferred first-line option across SARD-ILDs, while rituximab and cyclophosphamide remain therapeutic alternatives depending on disease subtype, severity, prior treatment history, and patient-specific factors (76). Nintedanib is conditionally recommended as a first-line option in SSc-ILD and may also be considered in progressive fibrosing autoimmune ILD, whereas JAK inhibitors and calcineurin inhibitors have disease-specific roles, particularly in IIM-ILD (76). Accordingly, the redox- and Treg-directed approaches discussed below should be viewed as investigational or potentially adjunctive strategies rather than replacements for established SARD-ILD treatment.

### 5.1 Redox and metabolic modulation

Because Treg stability and suppressive function are closely linked to mitochondrial metabolism and redox homeostasis, modulating of oxidative stress and cellular metabolism has been proposed as a potential strategy to preserve Treg function (15, 41). Experimental observations further illustrate the sensitivity of Tregs to metabolic perturbation. For example, high-salt exposure has been associated with impaired mitochondrial respiration and acquisition of a dysfunctional, pro-inflammatory Treg phenotype (77). However, whether correction of these metabolic abnormalities can be therapeutically exploited in SARD-ILD remains unknown.

More direct evidence for redox modulation of Treg stability comes from experimental autoimmune disease. In the collagen-induced arthritis model, excessive ROS accumulation in Tregs was associated with PTEN oxidation, activation of the Akt/mTOR/STAT3 pathway, SOCS3 upregulation, reduced Foxp3 expression, and impaired Treg stability. Antioxidant treatment with N-acetyl-L-cysteine (NAC) or TEMPOL attenuated several of these abnormalities, supporting a redox-sensitive mechanism regulating Treg homeostasis (16). Importantly, this evidence was obtained in an experimental autoimmune arthritis model rather than in pulmonary fibrosis or SARD-ILD.

Importantly, mechanistic evidence supporting antioxidant modulation of Treg biology should not be equated with clinical antifibrotic efficacy. In the PANTHER-IPF trial, the combination of prednisone, azathioprine, and NAC was terminated early due to increased mortality and hospitalization compared with placebo; however, these findings cannot be attributed specifically to NAC as it was administered as part of a three-drug regimen (78). In the subsequently completed NAC-monotherapy arm of PANTHER-IPF, NAC did not significantly reduce the decline in forced vital capacity compared with placebo over 60 weeks and provided no significant overall clinical benefit in patients with mild-to-moderate IPF (79). Although IPF differs biologically from SARD-ILD and these studies did not evaluate Treg-directed effects, they demonstrate that antioxidant activity alone does not necessarily translate into clinical antifibrotic benefit. Therefore, redox-directed therapies for SARD-ILD should be considered investigational and require disease-specific validation.

Mitochondria-targeted redox modulation has also been explored in experimental settings outside the autoimmune lung field. For example, mitochondrial oxidative stress was reduced by SS31 in an Alzheimer’s disease model (80). However, because that study did not investigate Tregs, it provides only indirect evidence for the therapeutic framework considered here. More broadly, ROS are not uniformly detrimental, and their biological effects depend on cellular source, magnitude, duration, and tissue microenvironment. Thus, antioxidant activity should not be equated with restoration of Treg function or clinical antifibrotic efficacy. Currently, redox-directed approaches remain mechanistically plausible but clinically unproven for Treg-targeted treatment of SARD-ILD. This level of evidence is substantially weaker than that supporting established immunomodulatory or antifibrotic therapies for selected SARD-ILD phenotypes (76).

### 5.2 Low-dose IL-2 and Treg expansion

Foxp3^+^ Tregs depend on IL-2 signaling for their survival, homeostasis, and suppressive function (28), providing a biological rationale for low-dose IL-2 therapy. In SLE, acquired IL-2 deficiency has been proposed to contribute to defective Treg homeostasis, and low-dose IL-2 was therefore developed as an immune-regulatory strategy aimed at preferentially restoring the Treg compartment rather than broadly suppressing immune responses (81). Early clinical studies showed that low-dose IL-2 could selectively correct quantitative and phenotypic Treg abnormalities in patients with SLE (82).

More recent clinical studies have provided additional evidence for the biological effects of low-dose IL-2. In the multicenter, double-blind, randomized phase II LUPIL-2 trial, low-dose IL-2 was generally well tolerated and effectively expanded Treg populations; however, the prespecified primary clinical endpoint was not met in the intention-to-treat population (83). A subsequent study further demonstrated that IL-2 immunotherapy expands human Treg subsets with distinct functional and tissue-homing characteristics (84). Collectively, these findings support a reproducible biological effect of low-dose IL-2 on human Tregs, but they do not establish therapeutic efficacy in SARD-ILD.

Low-dose IL-2-mediated Treg expansion has also been investigated in other immune-mediated diseases, including chronic graft-versus-host disease (GVHD), HCV-associated vasculitis, and type 1 diabetes (85–88). In chronic graft-versus-host disease, expanded Tregs retained regulatory characteristics and suppressive activity (85), providing evidence that IL-2 can promote functional rather than merely phenotypic Treg expansion. Nevertheless, these studies were conducted in substantially different disease and tissue environments, and their findings remain extrapolative when considering SARD-ILD.

Evidence directly related to pulmonary fibrosis remains predominantly experimental. In the Fra-2 transgenic mouse model, low-dose IL-2 treatment and/or adoptive Treg transfer restored Treg homeostasis and attenuated pulmonary fibrosis and vascular remodeling (36). In another murine study, Tregs limited pneumococcus-induced exacerbation of established pulmonary fibrosis (89). Importantly, this study addressed infection-triggered worsening of pre-existing fibrosis rather than the initiation or chronic progression of pulmonary fibrosis itself.

These findings should also be interpreted in light of the context-dependent roles of Tregs discussed above. Expansion of Tregs cannot automatically be assumed to produce an antifibrotic effect, as Treg-derived mediators may participate in both immune regulation and tissue remodeling depending on disease stage and the local microenvironment. Although low-dose IL-2 was generally well tolerated in SLE trials (83), its safety has not been established in patients with SARD-ILD receiving background immunosuppressive therapy. Future SARD-ILD studies should monitor infections and off-target expansion of other IL-2-responsive immune-cell populations, as well as the theoretical possibility that Treg expansion could enhance TGF-β- or Areg-associated remodeling in selected fibrotic microenvironments (24, 83). Therefore, although low-dose IL-2 is among the best-characterized pharmacological approaches for expanding human Tregs, its therapeutic relevance to SARD-ILD remains unproven. Accordingly, low-dose IL-2 should currently be considered an investigational adjunct rather than an alternative to established disease-modifying therapy for SARD-ILD (76).

### 5.3 JAK/TYK2 pathway modulation

JAK/STAT signaling regulates multiple inflammatory cytokine pathways and T-cell differentiation programs and represents an established therapeutic target in several immune-mediated diseases (90, 91). However, the effects of JAK inhibition on Treg biology are complex and should not be interpreted as uniformly beneficial.

More selective modulation of JAK-family signaling may exert distinct effects on Treg biology. Experimental evidence indicates that TYK2 inhibition can enhance Treg differentiation and suppressive function while simultaneously limiting Th1 and Th17 differentiation (92). These findings suggest that selective TYK2 inhibition may favor a more regulatory T-cell environment. However, the available evidence remains largely mechanistic, and direct therapeutic effects on pulmonary fibrosis or human SARD-ILD have not been established.

Thus, conventional JAK inhibition and selective TYK2 inhibition should not be considered mechanistically equivalent. Moreover, clinical benefits of JAK inhibitors in individual SARD-ILD subtypes should not automatically be attributed to restoration of Treg function, as these agents broadly modulate cytokine signaling. Importantly, JAK inhibitors are already conditionally recommended as a treatment option in IIM-ILD, reflecting disease-specific clinical use that is considerably more advanced than the Treg-directed mechanistic rationale discussed here (76).

### 5.4 Treg-based and metabolic engineering strategies

Direct manipulation of Treg metabolism or cellular properties represents another potential therapeutic avenue. In experimental graft-versus-host disease, genetic or pharmacological inhibition of acetyl-CoA carboxylase 1 (ACC1) enhanced Treg mitochondrial metabolism and suppressive capacity, thereby improving the therapeutic efficacy of adoptively transferred Tregs (15). These findings demonstrate that Treg metabolism can be therapeutically manipulated, although they remain extrapolative with respect to SARD-ILD.

Adoptive Treg therapy has progressed from experimental models to clinical evaluation in transplantation settings. Donor Treg therapy has shown feasibility and potential clinical benefit in the prevention of graft-versus-host disease (93); however, treatment context remains critical. In a randomized study, a Treg-containing graft administered without tacrolimus prophylaxis was associated with poorer control of acute GVHD compared with the same approach combined with tacrolimus (94), indicating that Treg therapy cannot necessarily replace established immunosuppressive treatment.

These limitations have motivated the development of engineered Tregs. TCR- or chimeric antigen receptor (CAR)-modified Tregs may offer improved antigen or tissue specificity compared with polyclonal Treg approaches, while additional engineering strategies aim to improve lineage stability, persistence, migratory capacity, and tissue adaptation (95, 96). Nevertheless, engineered Treg therapy remains at an early translational stage, and there is currently no established role for this approach in SARD-ILD.

Several additional challenges are particularly relevant to fibrotic lung disease, including incomplete knowledge of disease-specific antigens, uncertain pulmonary trafficking, concern regarding lineage stability, manufacturing complexity, and the possibility that enhanced tissue-repair programs may exert context-dependent pro-remodeling effects. These issues require direct evaluation in disease-specific pulmonary models before clinical translation can be considered. Given this early translational stage, adoptive or engineered Treg approaches currently have a substantially lower level of disease-specific evidence than established immunomodulatory therapies used in SARD-ILD (76).

### 5.5 Disease-specific translation and current therapeutic context

The therapeutic relevance of redox and Treg biology is unlikely to be uniform across SARD-ILD subtypes. SSc-ILD and anti-MDA5^+^ dermatomyositis-associated ILD illustrate this disease-specific heterogeneity. SSc-ILD and anti-MDA5^+^ DM-ILD are presented here as illustrative examples because disease- and lung-compartment-specific data are relatively more developed in these settings; they should not be considered representative of all SARD-ILD subtypes. In experimental pulmonary fibrosis, DUOX1-derived oxidative signaling has been shown to sustain TGF-β1 signaling and promote fibroblast activation and lung fibrosis (97). However, this study did not establish a direct DUOX1–Treg pathway or specifically demonstrate this mechanism in human SSc-ILD.

Human SSc-ILD studies have identified disease-associated pulmonary molecular programs (98) and profibrotic macrophage populations (99), supporting a multicellular model of fibrotic progression. These findings indicate that Treg dysregulation should be considered alongside fibroblast-, macrophage-, vascular-, and other immune-cell pathways rather than as an isolated therapeutic target. The relationship between these SSc-ILD-specific pathways and Treg dysfunction remains to be directly established.

In clinical practice, treatment of SSc-ILD is currently based primarily on immunomodulatory and antifibrotic approaches rather than direct manipulation of Tregs. Mycophenolate mofetil (MMF) and cyclophosphamide are supported by randomized trial evidence in SSc-ILD. In Scleroderma Lung Study II, both MMF and oral cyclophosphamide were associated with improvement in lung function and clinical outcomes, with MMF generally preferred due to its more favorable tolerability profile (100). Nintedanib provides a complementary antifibrotic strategy; in the SENSCIS trial, nintedanib significantly reduced the annual rate of FVC decline in patients with SSc-ILD compared with placebo (101). Additionally, in the autoimmune disease-related ILD subgroup of the INBUILD trial, nintedanib reduced the rate of FVC decline compared with placebo in patients with a progressive fibrosing phenotype (102). Tocilizumab has also been associated with preservation of lung function in selected patients with early inflammatory systemic sclerosis, although the primary skin endpoint of the phase III trial was not met (103). These established or clinically supported treatments have substantially stronger disease-specific clinical evidence than direct redox- or Treg-targeted approaches.

Therapeutic effects observed in one fibrotic disease should also not be assumed to translate directly to another. Pirfenidone, although used as an antifibrotic treatment in IPF, exacerbated Th2-driven vasculopathy and pulmonary abnormalities in an experimental model of SSc-ILD (104). This finding underscores the importance of disease-specific biological context and argues against extrapolating antifibrotic efficacy solely on the basis of shared fibrotic features. Consistent with this uncertainty, current ACR/CHEST guidance conditionally recommends against pirfenidone as a first-line treatment for SARD-ILD (76).

Anti-MDA5^+^ dermatomyositis-associated ILD represents a distinct immune-inflammatory phenotype with a high risk of rapidly progressive ILD. Single-cell and immune-profiling studies have identified prominent abnormalities involving multiple adaptive and innate immune populations, including activated T-cell and B-cell responses as well as interferon-associated immune programs (105–107). However, these studies do not establish Treg dysfunction as the primary driver of disease.

Clinical evidence for JAK inhibitor-based treatment is emerging in anti-MDA5^+^ dermatomyositis-associated rapidly progressive ILD. A recent real-world study reported improved outcomes when protein A immunoadsorption was combined with JAK inhibitor-based therapy (108). This disease-specific positioning aligns with current SARD-ILD treatment guidance, which conditionally supports JAK inhibitors in IIM-ILD, whereas such treatment is not recommended uniformly across all SARD-ILD subtypes (76). Nevertheless, these clinical observations do not establish that the therapeutic effect is mediated specifically through restoration of Treg function.

Rituximab and cyclophosphamide also provide an important clinical benchmark for emerging immune-directed strategies. In the RECITAL phase IIb trial of severe or progressive connective-tissue-disease-associated ILD, both rituximab and intravenous cyclophosphamide were associated with improvement in lung function; however, rituximab did not demonstrate superiority over cyclophosphamide for the primary endpoint (109). These results further illustrate that emerging Treg-directed interventions should be evaluated against active disease-modifying comparators rather than against the absence of treatment.

Taken together, these disease-specific observations argue against a uniform Treg-targeted treatment strategy across SARD-ILD. Redox modulation, low-dose IL-2, selective TYK2 inhibition, metabolic manipulation, and engineered Treg approaches currently represent therapeutic strategies with substantially different levels of evidence. They should therefore be considered investigational or potentially adjunctive approaches rather than replacements for established disease-specific immunomodulatory and antifibrotic treatment. A comparative assessment of the evidence level, translational position, and key limitations of these therapeutic strategies is summarized in **Supplementary Table 2**.

## 6 Challenges and future directions

A major challenge in defining the role of Tregs in SARD-ILD is the limited availability of disease-relevant human lung samples. Peripheral blood measurements may not faithfully reflect immune-cell composition, activation states, or regulatory programs within the diseased lung. This compartmental difference has been demonstrated directly in severe anti-MDA5^+^ dermatomyositis-associated ILD, in which paired single-cell RNA sequencing of bronchoalveolar lavage fluid (BALF) and peripheral blood revealed markedly different pulmonary and circulating immune landscapes (110). These findings emphasize the necessity of distinguishing systemic immune abnormalities from lung-localized processes when interpreting Treg dysregulation in SARD-ILD.

Direct analysis of human lung tissue is therefore particularly informative. Single-cell RNA sequencing of lung explants from patients with SSc-ILD demonstrated increased proportions of Tregs together with activated CD8^+^ tissue-resident memory T cells and NK-cell populations compared with healthy control lungs (111). Importantly, increased Treg abundance does not necessarily indicate preserved suppressive function and may instead reflect compensatory recruitment, altered tissue retention, or phenotypic shifts. Future studies should therefore distinguish Treg abundance from phenotype, lineage stability, and functional suppressive capacity.

BALF provides a less invasive approach for studying the pulmonary immune compartment, although it primarily samples cells within accessible airway and alveolar spaces and may incompletely represent immune populations embedded within fibrotic interstitium. Recent studies combining BALF single-cell profiling with paired blood analysis or clinical phenotyping in anti-MDA5^+^ DM-ILD and SSc-ILD demonstrate the feasibility of compartment-resolved immune analysis (110, 112). Ideally, future studies should integrate peripheral blood, BALF, and, when clinically available, lung tissue from the same patients and across longitudinal disease stages.

Spatial organization represents another potentially important but insufficiently studied dimension of Treg biology. In an experimental murine lung-transplantation model, Foxp3^+^ Tregs were found to persist in peribronchial and peribronchiolar regions and to form organized lymphoid clusters following CD25-biased IL-2 treatment (113). Although this model does not recapitulate SARD-ILD, it illustrates why spatial transcriptomics, multiplex imaging, and related approaches may provide information beyond conventional cell-frequency measurements. Whether comparable Treg niches exist in human SARD-ILD lungs remains unknown.

Functional interpretation remains an additional challenge. Treg-associated cytokines, Foxp3 expression, or cell abundance should not be considered interchangeable measures for suppressive function. In a murine model of bacterium-triggered acute exacerbation of pulmonary fibrosis, IL-17 deficiency was accompanied by enhanced pulmonary Treg responses, increased TGF-β, and attenuated inflammation and fibrosis (38); however, these parallel changes do not establish TGF-β as a direct surrogate of Treg function. Future studies should integrate transcriptional and phenotypic profiling with lineage-stability markers and functional suppression assays whenever feasible.

Disease heterogeneity further limits generalization across SARD-ILD. Human lung tissue evidence is currently concentrated mainly in SSc-ILD, severe anti-MDA5^+^ DM-ILD, and RA-ILD. Single-cell analysis of SSc-ILD lung explants identified increased Treg abundance, but did not assess suppressive function or demonstrate a mechanistic relationship with oxidative stress (111). Paired BALF and peripheral-blood profiling in severe anti-MDA5^+^ DM-ILD (110) and lung-explant profiling in RA-ILD (114) revealed disease- and tissue-specific immune programs without establishing a complete oxidative stress–Treg–fibrosis pathway. In antineutrophil cytoplasmic antibody-associated vasculitis, including microscopic polyangiitis, oxidative stress has been associated with instability of circulating Tregs; however, pulmonary Tregs and MPA-ILD were not specifically investigated (115). Direct lung-level evidence remains particularly limited in SjD-ILD, MPA-ILD, and MCTD-ILD. These absences should be interpreted as evidence gaps rather than as evidence against involvement of the pathway. Accordingly, the proposed framework may represent a potentially convergent but subtype-dependent hypothesis rather than a unified mechanism established across all SARD-ILDs (**Supplementary Table 1**).

Clinical translation also requires standardization of oxidative-stress biomarker assessment. Candidate measures, including 8-isoprostane, 8-hydroxy-2′-deoxyguanosine, 4-hydroxynonenal adducts, and the reduced-to-oxidized glutathione ratio, may vary according to specimen type, collection and storage conditions, assay platform, smoking, infection, oxygen exposure, and concurrent treatment (13, 14). Future studies should use harmonized sampling and analytical protocols, prespecified time points, and longitudinal validation against pulmonary outcomes such as FVC, DLCO, HRCT progression, and mortality rather than relying on isolated cross-sectional measurements.

Finally, the central unresolved question remains whether Treg dysregulation acts as a causal driver of pulmonary fibrosis, serves as a compensatory response to ongoing inflammation and tissue injury, or encompasses both roles in a stage-dependent manner. Addressing this question will require longitudinal human studies that integrate oxidative-stress biomarkers with compartment-specific Treg phenotyping, lineage-stability assessment, spatial localization, and clinical pulmonary outcomes. Such investigations are critical to determine which components of the proposed oxidative stress–Treg framework are directly operative in human SARD-ILD and which primarily reflect mechanisms extrapolated from experimental models or non-pulmonary disease contexts.

## 7 Conclusions

Although current evidence supports a potential mechanistic link among oxidative stress, Treg dysregulation, and pulmonary fibrotic remodeling in SARD-ILD, a unified causal oxidative stress–Treg pathway applicable across all SARD-ILD subtypes has not been established. Direct evidence from human lung is beginning to emerge; for instance, single-cell analysis of SSc-ILD lung explants identified increased proportions of Tregs together with activated CD8^+^ tissue-resident memory T cells and NK-cell populations (111). Nevertheless, altered Treg abundance alone does not establish impaired suppressive function or a causal role in fibrotic progression.

Mechanistically, available studies suggest that oxidative stress may influence Treg biology through redox-sensitive regulation of Foxp3 expression and stability, via metabolic and epigenetic pathways, through IL-2/STAT5-dependent regulation, and by remodeling of the local inflammatory microenvironment. However, many individual links within this framework remain supported primarily by experimental models, related autoimmune diseases, or other inflammatory settings rather than derived from direct mechanistic studies in human SARD-ILD. Furthermore, Tregs themselves should also not be regarded as uniformly antifibrotic. Preserved regulatory activity may limit excessive inflammatory injury, whereas selected tissue-repair programs may contribute to fibroblast activation and remodeling under specific conditions. Consequently, the net effect of Tregs is likely to depend on disease stage, tissue compartment, local microenvironment, and Treg functional state.

Emerging lung-level studies further emphasize both tissue and disease specificity. In severe anti-MDA5^+^ dermatomyositis-associated ILD, paired single-cell analysis demonstrated marked differences between pulmonary and peripheral immune states (110), while direct analysis of SSc-ILD lung tissue identified alterations in local Treg and other lymphoid populations (111). Similarly, single-cell analysis in RA-ILD has revealed lung-specific immune-cell programs that differ from those observed in other tissue compartments (114). Collectively, these findings argue against the assumption that mechanisms identified in one SARD-ILD subtype or derived solely from peripheral blood can be generalized across all SARD-ILDs, particularly SjD-ILD, MPA-ILD, and MCTD-ILD, for which direct lung-level evidence remains sparse.

From a therapeutic perspective, current management of SARD-ILD remains disease- and phenotype-specific, relying primarily on established immunomodulatory agents and, in selected fibrosing phenotypes, antifibrotic therapies (76). Redox modulation, low-dose IL-2, selective TYK2 inhibition, metabolic manipulation, and Treg-based cellular approaches remain at substantially different and generally earlier stages of translational development. Accordingly, these strategies should currently be regarded as investigational or potentially adjunctive rather than as replacements for established SARD-ILD treatment.

The central unresolved question is whether specific forms of Treg dysregulation act as causal drivers of pulmonary fibrosis, represent compensatory responses to ongoing inflammation and tissue injury, or encompass both roles in a context-dependent manner. Future longitudinal studies should integrate oxidative-stress biomarkers with lung-compartment-specific assessment of Treg abundance, phenotype, lineage stability, suppressive function, spatial localization, and pulmonary outcomes. Such investigations will be essential to distinguish established mechanisms from indirect associations and to determine whether the proposed oxidative stress–Treg framework can ultimately support biomarker-guided or therapeutic strategies in SARD-ILD.
