# Supplementary material for: Treg dysregulation in the oxidative lung milieu: an emerging mechanistic link in interstitial lung disease associated with systemic autoimmune rheumatic diseases
Source: Front Immunol. 2026 Sep 24;17:1882853. doi: 10.3389/fimmu.2026.1882853 (PMC13647569; doi:10.3389/fimmu.2026.1882853)
Supplement: Supplementary file 1 [file SupplementaryFile1.docx]

**Supplementary Table 1. Evidence hierarchy for the proposed oxidative stress-Treg-fibrosis framework in SARD-ILD.**

| **Mechanistic link** | **Evidence source/model** | **Evidence tier** | **Relevance to SARD-ILD** | **Key limitation** | **References** |
| --- | --- | --- | --- | --- | --- |
| Altered pulmonary Treg abundance and broader immune-cell states | Human lung tissue or BALF from SSc-ILD, anti-MDA5⁺ DM-ILD, and RA-ILD | Tier 1: direct human SARD-ILD evidence (observational) | Direct disease- and tissue-compartment evidence | Refs. 111 and 114 directly report increased pulmonary Treg proportions, whereas ref. 110 primarily characterizes broader immune-cell programs. Treg suppressive function and causality remain unproven. | Shi et al. (110); Padilla et al. (111); Suga et al. (114) |
| Treg deficiency → lung fibrosis and vascular remodeling | Fra-2 systemic-sclerosis model | Tier 1: disease-specific model | Direct disease-relevant preclinical evidence | Animal model; no human causal validation | Frantz et al. (36) |
| Oxidative stress → pulmonary injury and fibrosis | IPF and experimental pulmonary-fibrosis studies; DUOX1 lung-fibrosis model | Tier 2: indirect pulmonary | Supports pulmonary redox-fibrosis plausibility | Not a direct Treg mechanism in human SARD-ILD | Otoupalova et al. (13); Cheresh et al. (14); Louzada et al. (97) |
| ROS → PTEN oxidation → Akt/mTOR–STAT3 → SOCS3 upregulation → Treg instability | Collagen-induced arthritis model | Tier 2: other SARD model | Supports autoimmune redox-sensitive Treg dysregulation | Not demonstrated in lung or human SARD-ILD | Satooka et al. (16) |
| Treg-derived IL-10 →Mo-AM maturation | Experimental pulmonary fibrosis | Tier 2: indirect pulmonary | Lung-specific Treg-macrophage mechanism | Human SARD-ILD validation is absent | Farhat et al. (25) |
| Context-dependent Treg or Th17 repair programs modulate fibrotic remodeling | Cardiac allograft, intestinal fibrosis, and chronic pancreatitis | Tier 2: other-organ fibrosis | Supports context-dependent Areg and type-2 immune remodeling | Organ-specific evidence; pulmonary relevance uncertain | Warunek et al. (24); Zhao et al. (72); Glaubitz et al. (75) |
| Reduced oleic acid availability or IL-2R nitration impairs Treg function | Multiple sclerosis and EAE | Tier 3: extrapolated | Provides metabolic and IL-2R mechanistic clues | Non-SARD and non-pulmonary context | Pompura et al. (41); Wu et al. (64) |
| Mitochondrial oxidative stress and lipid peroxidation | Alzheimer's disease and other non-immune cellular models | Tier 3: extrapolated | Supports general redox and mitochondrial plausibility | Does not establish Treg dysfunction in SARD-ILD | Li et al. (42); Park et al. (43); Calvo-Rodriguez et al. (80) |
| Redox and stress-related milieus alter T-cell states and context-associated Treg abundance or survival | Tumor and chronic-stress models | Tier 3: extrapolated | Provides microenvironmental and telomere-damage clues | Unrelated disease settings; direct SARD-ILD relevance unconfirmed | Yu et al. (65); Zhang et al. (67); Hu et al. (68); Zhang et al. (69); Wesolowski et al. (70); Mitra et al. (71) |
| Subtype- and compartment-specific evidence | Human SSc-ILD, anti-MDA5⁺ DM-ILD, and RA-ILD studies; circulating AAV data | Mixed: direct pulmonary and indirect systemic evidence | Supports a subtype-dependent rather than uniform SARD-ILD framework | Direct lung-level evidence remains sparse for SjD-ILD, MPA-ILD, and MCTD-ILD; the complete pathway is unproven | Shi et al. (110); Padilla et al. (111); Suga et al. (114); Shimojima et al. (115) |

Tier 1 denotes evidence from human SARD-ILD or disease-specific SARD-ILD models, but not necessarily direct proof of the complete oxidative stress–Treg–fibrosis pathway. Tier 2 denotes indirect evidence from other SARDs, pulmonary fibrosis, or fibrosis in other organs. Tier 3 denotes extrapolated evidence from unrelated diseases, tissues, or experimental systems. Evidence tier indicates proximity to SARD-ILD, not methodological quality.

**Supplementary Table 2. Translational assessment of therapeutic strategies related to redox and Treg biology in SARD-ILD.**

| **Agent/strategy** | **Molecular target** | **Evidence source/model** | **Development stage** | **Position in SARD-ILD** | **Key limitation or safety concern** | **References** |
| --- | --- | --- | --- | --- | --- | --- |
| NAC or TEMPOL | ROS scavenging and redox homeostasis | CIA model; PANTHER-IPF trial reports | NAC/TEMPOL: preclinical autoimmune; NAC: clinical IPF | Investigational; no proven SARD-ILD benefit | In IPF, prednisone–azathioprine–NAC triple therapy increased mortality and hospitalization; NAC monotherapy showed no significant FVC benefit. | Satooka et al. (16); IPF Clinical Research Network et al. (78, 79) |
| SS31 | Mitochondrial ROS | Alzheimer’s disease mouse model | Preclinical; extrapolated | Very low relevance | No direct Treg, pulmonary fibrosis, or SARD-ILD evidence | Calvo-Rodriguez et al. (80) |
| Low-dose IL-2 | IL-2/STAT5 signaling and Treg expansion | SLE clinical studies; Fra-2 SSc model; contextual pulmonary-fibrosis and other-organ Treg-remodeling studies | Clinical in SLE; preclinical pulmonary evidence | Investigational adjunct | LUPIL-2 was well tolerated, but its primary endpoint was not met; SARD-ILD-specific safety and net remodeling effects remain uncertain; potential pro-remodeling effects of expanded Tregs are theoretical and context-dependent. | Warunek et al. (24); Frantz et al. (36); Humrich and Riemekasten (81); von Spee-Mayer et al. (82); Humrich et al. (83); Raeber et al. (84); Moyé et al. (89) |
| JAK inhibitors | Broad JAK/STAT cytokine signaling | IIM-ILD treatment guideline; anti-MDA5⁺ DM-RPILD real-world combination study involving a JAK inhibitor and protein A immunoadsorption | Disease-specific clinical use | Conditionally recommended in IIM-ILD | Broad immunomodulation; benefit from combination therapy cannot be attributed specifically to JAK inhibition or Treg restoration | Johnson et al. (76); Hu et al. (90); Shawky et al. (91); Fu et al. (108) |
| Selective TYK2 inhibition | TYK2; Th1/Th17 suppression and Treg preservation/enhancement | Human T-cell in vitro and IBD ex vivo models | Preclinical | Low; investigational | No direct pulmonary-fibrosis or human SARD-ILD evidence | Tuomela et al. (92) |
| ACC1 inhibition | FAO/OXPHOS and Treg mitochondrial metabolism | GVHD model | Preclinical; extrapolated | Very low; investigational | No pulmonary or SARD-ILD validation | McDonald-Hyman et al. (15) |
| Adoptive or engineered Tregs | Treg replacement, antigen specificity, and tissue targeting | Transplantation/GVHD clinical studies and translational reviews | Early clinical outside SARD-ILD | Experimental | Unknown lung trafficking and target antigens in SARD-ILD; lineage stability, infection risk, manufacturing complexity, and potential pro-remodeling effects require evaluation. | Meyer et al. (93); Bader et al. (94); Ho et al. (95); Bittner et al. (96) |
| MMF, cyclophosphamide, rituximab, or tocilizumab | Established immunomodulatory pathways | Guidelines and randomized clinical trials | Clinical standard or disease-specific option | Current therapeutic benchmark | Selection depends on disease subtype, severity, comorbidity, prior therapy, and safety profile | Johnson et al. (76); Tashkin et al. (100); Khanna et al. (103); Maher et al. (109) |
| Nintedanib | Antifibrotic tyrosine-kinase inhibition | SENSCIS and INBUILD trials | Clinical | Clinically supported and conditionally recommended for selected SSc-ILD or progressive fibrosing SARD-ILD phenotypes | Does not directly target Tregs; gastrointestinal adverse effects and disease-specific selection | Johnson et al. (76); Distler et al. (101); Matteson et al. (102) |
| Pirfenidone | Antifibrotic signaling | Murine SSc-ILD model | Preclinical SSc-ILD; clinical use in IPF | Conditionally recommended against as first-line SARD-ILD therapy | Exacerbated Th2-driven vasculopathy and pulmonary abnormalities in the SSc-ILD model | Johnson et al. (76); Birnhuber et al. (104) |

References 24 and 89 provide contextual evidence regarding Treg-mediated remodeling or pulmonary fibrosis rather than direct evidence of low-dose IL-2 efficacy. Redox- or Treg-directed strategies remain investigational or potentially adjunctive and should not replace established disease-specific therapy. Evidence from IPF or non-SARD settings should not be directly generalized to SARD-ILD.
